# Corneal epithelial thickness can reflect the stage of Parkinson’s disease and the severity of dry eye

**DOI:** 10.3389/fnagi.2026.1737800

**Published:** 2026-03-25

**Authors:** Dongyu Li, Jiazheng Ji, Xinyu Zhang, Fan Yang, Xin Jin, Jingrao Wang, Shumin Li, Lifen Yao, Hong Zhang

**Affiliations:** 1 Eye Hospital, The First Affiliated Hospital of Harbin Medical University, Harbin, Heilongjiang, China; 2 Department of Neurology, First Affiliated Hospital of Harbin Medical University, Harbin, Heilongjiang, China

**Keywords:** anterior segment optical coherence tomography, corneal epithelial thickness, dry eye, H-Y stage, Parkinson’s disease

## Abstract

**Purpose:**

To systematically analyze corneal epithelial thickness (CET) distribution in Parkinson’s disease (PD) using anterior segment optical coherence tomography (AS-OCT) and investigate its correlation with disease stage and dry eye severity.

**Methods:**

This cross-sectional study enrolled 60 PD patients (120 eyes) and 29 age- and sex-matched healthy controls (58 eyes). The accessed parameters included dry eye disease (DED) metrics, such as the Ocular Surface Disease Index (OSDI), Schirmer I test (sIt), tear breakup time (BUT), corneal fluorescein staining (CFS), corneal sensitivity, and zonal CET measured by AS-OCT in central 2-mm, 5-mm, and 6-mm zones. Baseline PD characteristics included disease duration, levodopa equivalent daily dose (LEDD), Hoehn and Yahr (H-Y) stage, Movement Disorder Society Unified Parkinsond dPisease Rating Scale (MDS-UPDRS Part III), and Montreal Cognitive Assessment (MoCA).

**Results:**

PD patients showed significantly higher OSDI and CFS (*p* < 0.001), and lower sIt, BUT, and CET (*p* < 0.001). CET was significantly thinner in temporal, superior, and nasal quadrants of the 6-mm zone (*p* < 0.05). Temporal 6-mm CET was positively correlated with BUT (*p* = 0.024). With advancing H-Y stage, peripheral CET progressively thinned, while OSDI increased and BUT and sIt decreased, indicating worsening DED.

**Conclusion:**

This study demonstrates, for the first time, that CET correlates with both dry eye severity and H-Y stage in PD. AS-OCT-based CET mapping may serve as a non-invasive biomarker for early neurodegeneration detection and guide ocular surface management.

## 1 Introduction

PD is a neurodegenerative disorder characterized pathologically by the progressive degeneration of dopaminergic neurons in the substantia nigra. Its clinical phenotype encompasses four core motor symptoms (resting tremor, rigidity, bradykinesia, and postural instability) and a wide range of non-motor symptoms (Braak et al., 2003). Visual system involvement is prominent among non-motor symptoms, with epidemiological studies indicating visual dysfunction in approximately 60–80% of PD patients (Nieto-Escamez et al., 2023). PD-related visual impairments include DED, diplopia, reduced spontaneous blink rate (< 10 blinks/min), chronic blepharitis, blepharospasm, thinning of the retinal pigment epithelium, and impaired accommodation-convergence (Biousse et al., 2004; Weil, 2020). These pathological changes can lead to clinical syndromes, such as blurred vision, reduced reading endurance, and visual hallucinations, significantly impacting vision-related quality of life (VR-QOL) (Morthen et al., 2022).

DED is a multifactorial chronic ocular surface disease defined by the loss of tear film homeostasis, involving tear deficiency or excessive evaporation, ocular surface inflammation, and neurosensory abnormalities. Clinical manifestations include ocular dryness, foreign body sensation, visual fluctuation, and shortened BUT, often accompanied by corneal or conjunctival epithelial damage (Craig et al., 2017). Notably, PD-related dopaminergic degeneration extends to the trigeminal-somatosensory pathway, impairing corneal nociception and blink reflex (Marino et al., 2020). This may disrupt tear film homeostasis, suggesting a shared neurodegenerative mechanism underlying both motor and ocular surface symptoms. A recent meta-analysis reported a pooled DED prevalence of 70% (95% CI: 64–76%) in PD patients, significantly higher than in age-matched controls (OR = 4.2, *p* < 0.001) (Nagino et al., 2022), highlighting the urgent need for ocular surface health management in this population.

AS-OCT enables non-contact, high-resolution imaging for precise quantification of CET distribution and identification of subclinical corneal structural changes (Gupta et al., 2022). Current studies have indicated that CET measurement holds value in suggesting early disease course and reflecting the severity of dry eye in certain conditions, such as Sjögren’s syndrome (Gupta et al., 2022) and evaporative dry eye associated with meibomian gland dysfunction (Abou Shousha et al., 2020). However, in PD, the characteristics of CET distribution and its correlation with disease stage and dry eye severity remain unexplored. Given the high prevalence of dry eye in PD patients and the critical importance of early diagnosis for optimal management, this case-control study aimed to systematically compare CET zonal differences (central 2-mm, paracentral 5-mm, and 6-mm zones) between PD patients and age- and sex-matched healthy controls, analyzing correlations with H-Y stage and key DED indicators (OSDI symptom score, Schirmer I test, BUT, CFS score). These findings may provide evidence for the early diagnosis and stratified management of PD-related ocular surface disorders.

## 2 Materials and methods

### 2.1 Participants

This cross-sectional observational study was conducted at the eye hospital of the First Affiliated Hospital of Harbin Medical University between June 2024 and February 2025. It was approved by the Ethics Committee of the First Affiliated Hospital of Harbin Medical University (IRB AF/SC-04/02.0) and conducted in accordance with the principles of the Declaration of Helsinki (2013 revision) (World Medical Association, 2013). All participants provided written informed consent for the use of clinical data in research analysis. Comprehensive assessments, including diagnosis confirmation, medical history evaluation, questionnaire completion, and neurological examinations, were performed by experienced neurologists for each participant. All participants were of Han Chinese ethnicity, recruited from Northeast China.

#### 2.1.1 Inclusion criteria

Recruitment of PD patients aged 50–80 years were conducted at the Department of Neurology, First Affiliated Hospital of Harbin Medical University, from June 2024 to February 2025. Inclusion criteria, patients were included in the PD group based on the following criteria; Patients aged 50–80 years diagnosed with “clinically established PD” or “linically probable PD” (Jankovic, 2008), capable of cooperating with ophthalmic examinations (including history taking and scale assessments). Control Group was included based on the following criteria; Age- and sex-matched healthy volunteers without PD symptoms or neurological signs, and with sIt ≥ 10-mm/5 min and BUT ≥ 10 s.

#### 2.1.2 Exclusion criteria

Exclusion criteria encompassed Other neurological disorders (e.g., corticobasal degeneration, dementia with Lewy bodies, Parkinson-plus syndromes), or secondary parkinsonism (drug-induced, vascular, infectious, traumatic); Ocular diseases (e.g., refractive media opacities, structural macular damage, glaucoma, uveitis, optic neuropathy, active intraocular inflammation); Systemic diseases (e.g., uncontrolled hypertension, diabetes, severe cardiopulmonary/hepatic/renal disease, malignancy, and Sjögren’s syndrome, which was screened using a symptom questionnaire for xerostomia and systemic manifestations, since it is associated with dry eye symptoms that could interfere with the study (Shiboski et al., 2017); medication-induced dry eye, ascertained through a detailed medication history. Patients were excluded if they were currently or recently using drugs known to exacerbate dry eye, such as anticholinergic medications (e.g., trihexyphenidyl, amantadine), antipsychotics and antidepressants (e.g., clozapine, selective serotonin reuptake inhibitors [SSRIs]), beta-blockers, or topical ophthalmic solutions containing preservatives such as benzalkonium chloride; Poor-quality OCT/OCTA images (signal strength < 7); Inability to cooperate due to PD-related movement disorders or facial anatomical abnormalities.

### 2.2 Data collection and assessment parameters

Clinical data comprised age, sex, disease duration, and education level. MDS-UPDRS Part III Score assessed 18 motor functions (bradykinesia, tremor, postural stability, gait) via clinical examination. Each item scored 0–4 (0 = normal; 4 = severe disability). Total score range 0–132; higher scores indicate worse motor dysfunction (Goetz et al., 2008).

#### 2.2.1 Parkinson’s disease mild cognitive impairment Screen (MoCA adapted for PD)

To account for the influence of educational attainment on cognitive performance, one point was added to the total score for individuals with ≤ 12 years of formal education. Assessed 8 cognitive domains (attention, executive function, memory, language, visuospatial ability, etc.). Total score 0–30; ≤ 25 suggests cognitive impairment. The PD-adapted version modified items to reduce motor dependency (Dalrymple-Alford et al., 2010).

#### 2.2.2 Levodopa equivalent daily dose

Calculated using standard conversion factors based on type and dose of anti-parkinsonian medications (Tomlinson et al., 2010).

#### 2.2.3 Hoehn-Yahr staging

Stage 1: Unilateral involvement only, minimal functional impairment. Stage 2: Bilateral involvement without impairment of balance. Stage 3: Mild to moderate bilateral disease with postural instability; physically independent. Stage 4: Severe disability; requires substantial assistance but can walk/stand unassisted. Stage 5: Wheelchair-bound or bedridden; requires constant nursing care (Hoehn and Yahr, 1967).

#### 2.2.4 Ocular surface disease index score

Dry eye symptoms were assessed using the standardized Ocular Surface Disease Index (OSDI) questionnaire. The OSDI comprises 12 items across three subscales: ocular symptoms (3 items: photophobia, foreign body sensation, and eye pain or soreness), visual function (6 items: blurred vision, poor vision, difficulty reading, difficulty driving at night, difficulty using a computer or gaming console, and difficulty watching television), and environmental triggers (3 items: discomfort in windy conditions, very dry environments, and air-conditioned rooms). Each item was rated on a 0–4 scale based on the frequency of symptoms experienced during the past week. The total OSDI score was calculated using the formula: (sum of scores for all answered items × 25)/(number of answered items), yielding a range from 0 to 100, with higher scores indicating greater severity of dry eye disease (Schiffman et al., 2000; The Ocular Surface, 2007).

#### 2.2.5 Schirmer I test

Measured basal tear secretion without topical anesthesia. Standardized filter strips (5-mm × 35-mm) were placed in the lateral third of the lower conjunctival fornix, and the wetting length (mm) was recorded after 5 min of eye closure (Valim et al., 2015).

#### 2.2.6 Corneal fluorescein staining score

Graded using the Oxford scale. The cornea was divided into 4 quadrants; each quadrant scored 0–3 (0 = no staining; 1 = 1–30 punctate dots; 2 = > 30 punctate non-confluent dots; 3 = confluent staining or ulcer). Total score range 0–12 (Bron et al., 2003).

#### 2.2.7 Break-up time

A core indicator for assessing tear film stability. After instillation of sodium fluorescein into the conjunctival sac, the patient is asked to blink naturally several times and then refrain from blinking. The time (in seconds) from the last complete blink to the first appearance of a randomly distributed dry spot on the corneal surface is observed under cobalt blue light using a slit lamp. A shortened BUT (typically ≤ 10 s) indicates tear film instability and is a key feature of evaporative or mixed dry eye (Tagawa et al., 2019).

#### 2.2.8 Corneal sensitivity

Quantitatively assessed using a Cochet-Bonnet aesthesiometer. This device applies a calibrated mechanical stimulus pressure via a nylon filament of varying length (0–60 mm), with shorter lengths corresponding to higher pressure. During measurement, the filament is applied perpendicularly to the corneal surface, from the periphery to the center, and the longest filament length (mm) at which the patient can reliably perceive the stimulus is recorded. A lower length value indicates reduced corneal sensitivity. Decreased corneal sensitivity suggests impaired afferent function of the trigeminal nerve pathway, commonly observed in diabetic neuropathy, post-laser refractive surgery, and neurodegenerative diseases including Parkinson’s disease (Benítez-Del-Castillo et al., 2007).

#### 2.2.9 Corneal epithelial thickness measurement

CET was measured using AS-OCT (ANTERION^®^). Scan parameters: axial resolution < 10 μm, 65 radial B-scans, 256 A-scans per B-scan, 7-mm scan diameter centered on the corneal apex, generating epithelial thickness maps (Zhou et al., 2021). CET was analyzed in the central 2-mm zone and the temporal (T), superior (S), nasal (N), and inferior (I) quadrants of the concentric 5-mm and 6-mm rings.

### 2.3 Statistical analysis

Data were analyzed using R software. Continuous variables are presented as mean ± standard deviation (SD) if normally distributed, or median (interquartile range, IQR) if non-normally distributed. Categorical variables are presented as frequencies (percentages). Group comparisons: Independent samples *t*-test (normally distributed data), Wilcoxon rank-sum test (non-normally distributed data), Chi-square test (categorical variables). Spearman’s rank correlation coefficient (ρ) assessed correlations. Multiple linear regression models evaluated associations between CET and clinical parameters (age, disease duration, LEDD, etc.). The significance level was set at α = 0.05 (two-tailed).

## 3 Results

Clinical and Demographic Profiles A total of 60 patients with PD were included in this study, comprising 34 females and 26 males, with a mean age of 64.02 ± 7.64 years. Additionally, the study involved 29 control subjects, consisting of 15 females and 14 males, who had a mean age of 62.35 ± 10.15 years. There were no significant differences in age (*P* = 0.425), gender (*P* = 0.561), and education (*P* = 0.859) between the PD group and the control group. Compared with the control group, the MoCA score of patients in the PD group was significantly reduced (*P* < 0.001) (Table 1).

**TABLE 1:** Comparison of basic information of participants.

Features	Control (*n* = 29)	PD (*n* = 60)	c2/t	*P*
Age, years	62.35 ± 10.15	64.02 ± 7.64	0.80564	0.425
Sex			0.33747	0.561
M	14	26		
F	15	34		
Education			1.3126	0.859
Primary school	4	4		
Junior high school	8	20		
High school	11	25		
Junior college	4	7		
University	2	4		
Disease duration, years	NA	5 (3, 9)		
LEDD, mg	NA	450(375, 700)		
MDS UPDRS III	NA	28(14, 46)		
H-Y stage	NA	2.30 ± 0.90		
MoCA	27 (26, 28)	25 (21, 27)	−5.0411	**<0.001**

Data with statistically significant differences (*p* < 0.05) are bolded.

### 3.1 Comparison of ocular surface parameters: PD group vs. control group

Ocular surface function was significantly worse in the PD group. The PD group showed markedly higher OSDI scores (median 52.27, IQR: 42.61–59.66) compared to controls (median 4.54, IQR: 0–15.91; *p* < 0.001, Wilcoxon test), reduced Schirmer I test values (10.18 ± 2.55 mm vs. 15.02 ± 1.74 mm; *p* < 0.001, *t*-test), shorter tear film breakup time (median 5.12 s, IQR: 4.01–5.99 vs. median 11.45 s, IQR: 10.64–12.46; *p* < 0.001), and greater corneal fluorescein staining (median 1, IQR: 0.5–1.5 vs. median 0, IQR: 0–0; *p* < 0.001). Furthermore, corneal sensitivity was significantly reduced in the PD group, both in the central zone (median 5.25 mm, IQR: 4.75–5.66 vs. 5.16 mm, IQR: 4.96–5.50) and peripheral zone (median 5.75 mm, IQR: 5.25–6.00 vs. 5.50 mm, IQR: 5.38–5.62; all *p* < 0.001). Complete data are presented in Table 2.

**TABLE 2:** Ocular surface data in eyes with PD and CON groups.

	Mean ± SD/M (25%, 75%)		*t/Z*	*P*
Features	PD	CON		
OSDI	52.27 (42.61, 59.66)	4.54(0, 15.91)	−7.09	**<0.001**
sIt(mm)	10.18 ± 2.55	15.02 ± 1.74	−9.22	**<0.001**
BUT(s)	5.12(4.01, 5.99)	11.45(10.64, 12.46)	−7.36	**<0.001**
CFS	1(0.5, 1.5)	0(0, 0)	−5.76	**<0.001**
Central cornea	5.25(4.75, 5.66)	5.75(5.25, 6)	−3.79	**<0.001**
Peripheral cornea	5.16(4.96, 5.5)	5.5(5.38, 5.62)	−3.57	**<0.001**

OSDI, Ocular Surface Disease Index; sIt, Schirmer I test (tear secretion); BUT, Tear breakup time; CFS: Corneal fluorescein staining score. Normally distributed data are presented as mean ± SD; multiple comparisons between groups used LSD-*t* test. Non-normally distributed data are presented as median [IQR; M (p25, p75)]. Multiple comparisons between groups were corrected using the Bonferroni method. Data with statistically significant differences (*p* < 0.05) are bolded.

### 3.2 Characteristics of corneal epithelial thickness distribution

AS-OCT measurements revealed significantly thinner CET in the PD group compared to controls in the temporal (T6 mm: 52.18 ± 2.47 μm vs. 53.43 ± 2.28 μm, *p* = 0.024), superior (S6 mm: 52.64 ± 2.53 μm vs. 54.06 ± 1.97 μm, *p* = 0.009), and nasal (N6 mm: 53.66 ± 2.50 μm vs. 54.96 ± 2.22 μm, *p* = 0.02) quadrants of the 6-mm zone, suggesting peripheral neurodegeneration may preferentially affect the corneal periphery. Complete data are presented in Table 3.

**TABLE 3:** Corneal epithelial in eyes with PD and CON groups.

	Mean ± SD		*t*	*p*
Epithelial thickness	PD	CON		
2 mm (μm)	53.42 ± 3.05	53.49 ± 2.55	−0.11	0.91
T5 mm (μm)	53.75 ± 3.01	54.25 ± 2.45	−1.08	0.284
S5 mm (μm)	52.08 ± 3.61	52.43 ± 1.80	−0.49	0.623
N5 mm (μm)	53.45 ± 3.11	54.01 ± 2.61	−0.84	0.406
I5 mm (μm)	53.58 ± 2.16	53.73 ± 1.15	−0.44	0.662
T6 mm (μm)	52.18 ± 2.47	53.43 ± 2.28	−2.29	**0.024**
S6 mm (μm)	52.64 ± 2.53	54.06 ± 1.97	−2.66	**0.009**
N6 mm (μm)	53.66 ± 2.50	54.96 ± 2.22	−2.37	**0.02**
I6 mm (μm)	54.05 ± 2.52	54.93 ± 2.26	−1.59	0.115

Normally distributed data are presented as mean ± SD. Multiple comparisons between groups were performed using LSD-*t*-test and corrected using the Bonferroni method. Data with statistically significant differences (*p* < 0.05) are bolded.

### 3.3 Correlation between CET and dry eye parameters in PD patients

Correlations between CET and DED parameters in PD patients are shown in Table 4.

**TABLE 4:** Correlation between epithelial and DED index.

Epithelial thickness		OSDI	BUT(s)	sIt(mm)	CFS
2 mm(μm)	*r*	−0.046	−0.021	−0.2	0.151
	*p*	0.671	0.844	0.06	0.158
T5 mm(μm)	*R*	−0.109	−0.004	−0.03	0.008
	*P*	0.311	0.968	0.78	0.94
S5 mm(μm)	*r*	0.049	0.185	−0.116	0.121
	*p*	0.647	0.082	0.281	0.257
N5 mm(μm)	*r*	−0.139	0.23	−0.033	0.057
	*p*	0.194	**0.03**	0.76	0.598
I5 mm(μm)	*r*	−0.009	0.178	−0.041	0.043
	*p*	0.934	0.095	0.704	0.691
T6 mm(μm)	*r*	−0.226	0.235	0.057	−0.077
	*p*	**0.033**	**0.027**	0.596	0.472
S6 mm(μm)	*r*	−0.17	0.265	0.175	−0.214
	*p*	0.111	**0.012**	0.102	0.044
N6 mm(μm)	*r*	−0.314	0.33	0.089	−0.078
	*p*	0.003	**0.002**	0.409	0.467
I6 mm(μm)	*r*	−0.039	0.189	−0.016	0.077
	*P*	0.714	0.076	0.88	0.475

OSDI, Ocular Surface Disease Index; sIt, Schirmer I test (tear secretion); BUT, Tear breakup time; CFS, Corneal fluorescein staining score. Spearman’s correlation coefficient (ρ) was used to evaluate linear correlations between CET (μm) and ocular surface parameters (OSDI, BUT, sIt, CFS). Data with statistically significant differences (*p* < 0.05) are bolded.

CET in the temporal, superior, and nasal quadrants of the concentric 6-mm zone showed significant positive correlations with BUT (*p* < 0.05).

**FIGURE 1:**
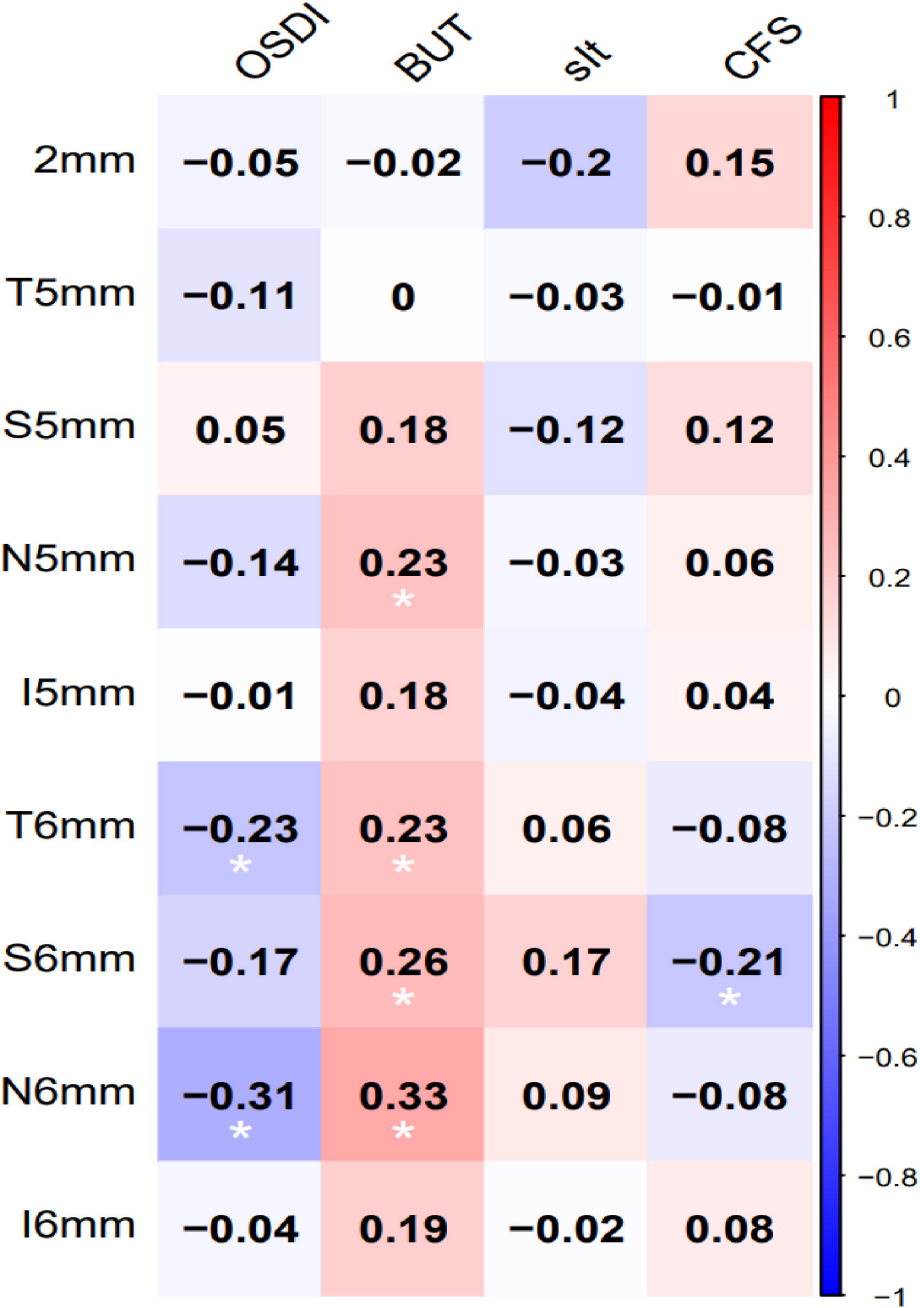
Heatmap of correlations between zonal CET and ocular surface parameters in PD patients. Correlation strength is indicated by color intensity, with redder huesrepresenting stronger positive correlations and bluer hues representing stronger negative correlations.

**FIGURE 2:**
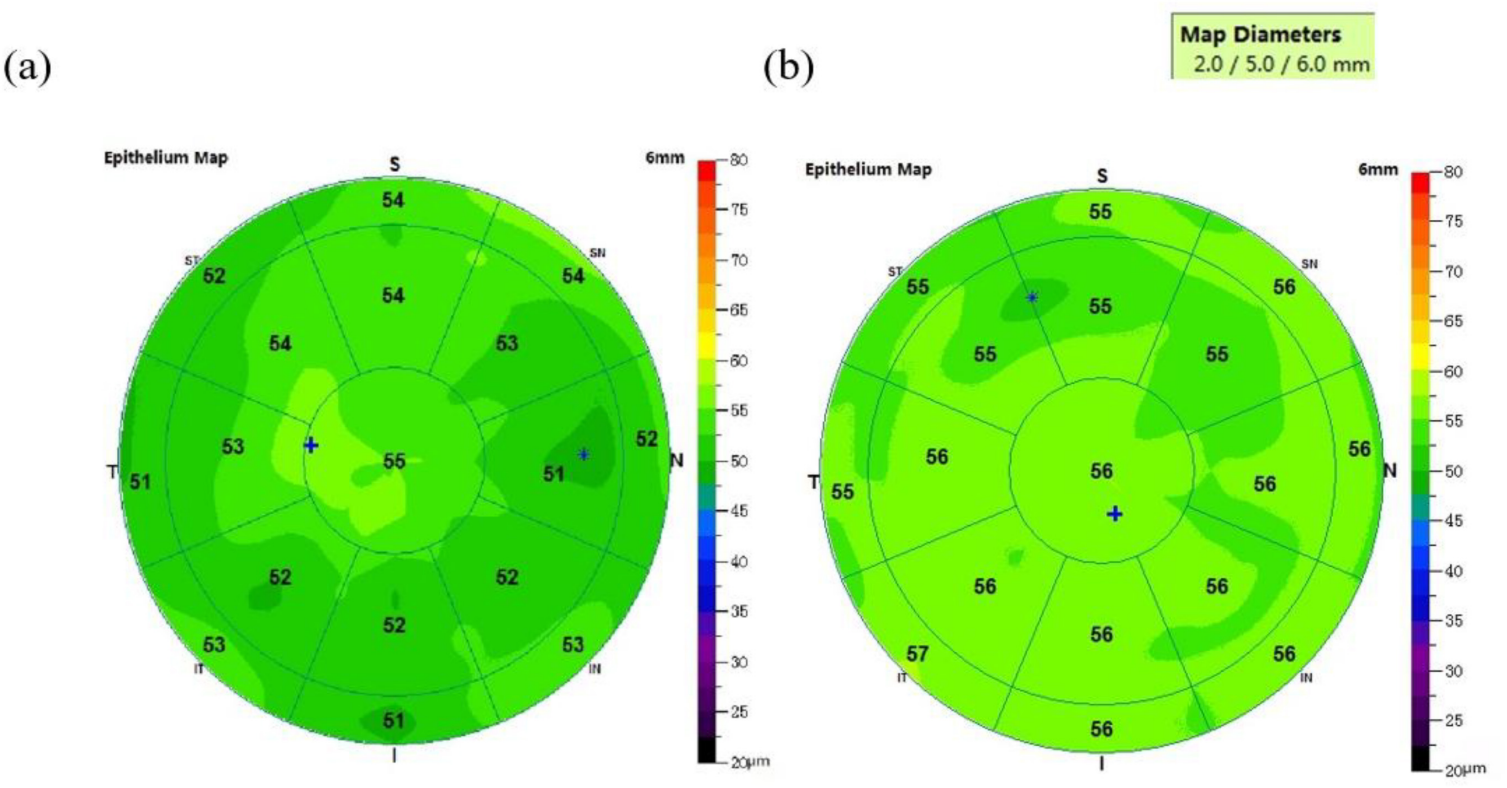
Comparison of corneal epithelial thickness map parameters between PD and CON. **(a)** The corneal epithelial thickness profile in the PD patient group, while **(b)** the corresponding profile in the control (CON) group. The scan radius was 6 mm.

Nasal 5-mm CET also correlated positively with BUT (ρ = 0.23, *p* = 0.03). Correlations between CET and DED parameters were generallysignificant in the concentric 6-mm zone (Figure 1). These results suggest corneal epithelial thinning may exacerbate tear film instability. In Figure 2, the PD group exhibited significantly thinner corneal epithelium in the peripheral 6-mm zone compared with the CON group.

### 3.4 Correlation analysis of ocular surface parameters with baseline data in PD Patients

**FIGURE 3:**
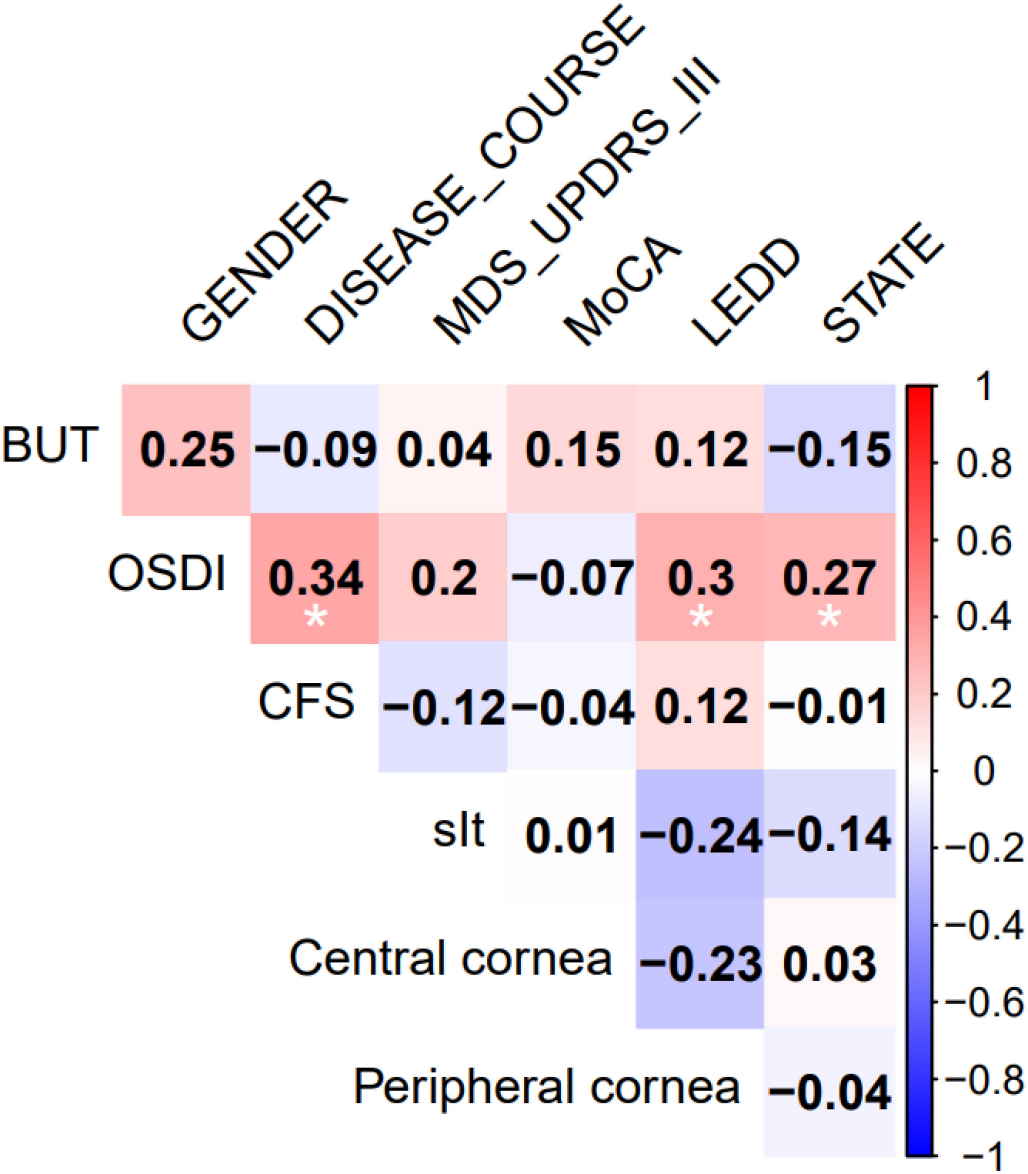
Heatmap of correlations between ocular surface parameters central corneal sensitivity, peripheral corneal sensitivity, and baseline data in PD patients. Correlation strength is indicated by color intensity, with redder hues representing stronger positive correlations and bluer hues representing stronger negative correlations.

Correlations between ocular surface parameters and baseline PD characteristics are presented in Table 5. OSDI scores showed significant positive correlations with PD disease duration, LEDD, and H-Y stage (Figure 3).

**TABLE 5:** Correlation between basic information and DED index.

Features		Age	Gender	Disease duration	MDS-UPDRS III	MoCA	LEDD	H-Y
BUT(s)	*r*	−0.001	0.246	−0.087	0.043	0.149	0.122	−0.153
	*p*	0.991	0.058	0.508	0.746	0.255	0.355	0.243
OSDI	*r*	0.121	0.141	0.344	0.196	−0.066	0.303	0.272
	*p*	0.356	0.283	**0.007**	0.134	0.618	**0.019**	**0.036**
CFS	*r*	0.031	−0.048	0.084	−0.119	−0.035	0.125	−0.006
	*p*	0.813	0.717	0.523	0.365	0.789	0.34	0.962
sIt(mm)	*r*	−0.073	0.027	−0.345	0.011	0.009	−0.245	−0.136
	*p*	0.579	0.835	**0.007**	0.931	0.944	0.059	0.298
Central cornea	*R*	0	−0.119	0.03	0.137	0.004	−0.228	0.027
	*P*	0.998	0.365	0.821	0.295	0.976	0.08	0.838
Peripheral cornea	*R*	−0.136	−0.078	−0.076	0.062	−0.073	−0.159	−0.042
	*P*	0.3	0.553	0.566	0.637	0.582	0.225	0.752

OSDI, Ocular Surface Disease Index; sIt, Schirmer I test (tear secretion); BUT, Tear breakup time; CFS, Corneal fluorescein staining score; MDS-UPDRS III, Movement Disorder Society-Unified Parkinson’s Disease Rating Scale Part III; MoCA, Montreal Cognitive Assessment adapted for PD; LEDD, Levodopa equivalent daily dose; H-Y, Hoehn-Yahr stage. Spearman’s correlation coefficient (ρ) was used to assess linear correlations between baseline data and dry eye indicators. Data with statistically significant differences (*p* < 0.05) are bolded.

### 3.5 Correlation between H-Y stage and CET distribution in PD patients

A clear trend of progressive corneal epithelial thinning was observed with advancing H-Y stages. Quantitative analysis demonstrated that from H-Y stage 1 to stage 3, the mean corneal epithelial thickness showed a consistent and gradual decrease across all quadrants of the paracentral 5-mm and peripheral 6-mm zones. This pervasive thinning pattern was evident in the superior, temporal, nasal, and inferior regions. In contrast, the central 2-mm zone maintained relative stability without a pronounced thinning trend across the same stages. A robust correlation was observed between advancing H-Y stage and progressive epithelial thinning across the corneal periphery. Complete data are presented in Table 6.

**TABLE 6:** Corneal epithelial thickness in H-Y stages.

	Mean ± SD			*F*	*P*
Epithelial thickness	1	2	3		
2 mm (μm)	53.94 ± 3.45	52.90 ± 2.96	53.73 ± 2.28	0.78	0.461
T5 mm (μm)	54.35 ± 2.96	53.59 ± 3.33	52.91 ± 1.99	0.86	0.428
S5 mm (μm)	53.40 ± 3.39	52.16 ± 2.09	51.02 ± 3.93	2.87	0.065
N5 mm (μm)	53.71 ± 3.41	53.54 ± 3.17	52.62 ± 2.25	0.43	0.650
I5 mm (μm)	54.29 ± 2.47	54.01 ± 2.43	53.18 ± 3.32	0.78	0.461
T6 mm (μm)	52.83 ± 2.44	52.04 ± 2.73	51.11 ± 1.18	1.79	0.176
S6 mm (μm)	53.30 ± 2.18	52.47 ± 2.84	52.18 ± 2.66	1.26	0.291
N6 mm (μm)	53.95 ± 2.89	53.37 ± 2.50	53.29 ± 1.51	0.27	0.765
I6 mm (μm)	54.61 ± 2.71	54.40 ± 2.22	53.58 ± 2.67	0.94	0.397

T, Temporal; S, Superior; N, Nasal; I, Inferior corneal quadrant. Numbers (e.g., 5 mm, 6 mm) indicate measurement diameter. Spearman’s rank correlation analysis was used for correlation assessment. *P* < 0.05 was considered significant.

### 3.6 Correlation between H-Y stage and ocular surface parameters in PD patients

DED severity progressively worsened with advancing H-Y stage (1–3). Corresponding changes in ocular surface parameters were observed: OSDI scores increased from stage 1 (median 46.59, IQR: 31.82–58.90) to stage 3 (median 62.50, IQR: 52.27–67.71), *p* = 0.099 (Wilcoxon test); sIt decreased from stage 1 (10.48 ± 2.57 mm) to stage 3 (9.30 ± 1.89 mm), *p* = 0.478; BUT decreased from stage 1 (median 5.63 s) to stage 3 (median 4.96 s), *p* = 0.496. Complete data are presented in Table 7.

**TABLE 7:** Ocular surface parameters in H-Y stages.

	Mean ± SD/M (25%, 75%)			*F/H*	*P*
Features	1	2	3		
OSDI	46.59(31.82, 58.90)	53.41(47.16, 59.09)	62.50(52.27, 67.71)	4.63	0.099
sIt(mm)	10.48 ± 2.57	10.25 ± 2.74	9.30 ± 1.89	0.75	0.478
BUT(s)	5.63(4.48, 6.14)	4.94(4.01, 5.87)	4.96(4.08, 5.39)	1.4	0.496
CFS	1.25(0.50, 1.50)	1(0.50, 1.62)	1(0.50, 1.38)	0.42	0.81
Central cornea	5.25(5.00, 5.72)	5.00(4.50, 5.50)	5.75(5.25, 5.75)	7.65	**0.022**
Peripheral cornea	5.25(5.00, 5.46)	5.19(4.81, 5.38)	5.16(4.98, 5.50)	0.41	0.815

OSDI, Ocular Surface Disease Index; sIt, Schirmer I test (tear secretion); BUT, Tear breakup time; CFS, Corneal fluorescein staining score. Normally distributed data are presented as mean ± SD; comparisons among three groups used one-way ANOVA, with multiple comparisons between groups using LSD-*t*-test. Non-normally distributed data are presented as median [IQR; M (p25, p75)]. Comparisons among three groups used Wilcoxon test, with multiple comparisons between groups corrected using the Bonferroni method. *P* < 0.05 was considered significant.

## 4 Discussion

### 4.1 Non-motor symptoms in PD and the emergence of dry eye

Parkinson’s disease has long been characterized by its cardinal motor symptoms; however, the significance of non-motor symptoms (NMS) is increasingly recognized as a major determinant of quality of life and overall disability. Recent studies have emphasized that non-motor manifestations often cause greater disability than motor symptoms themselves and require comprehensive management strategies (Barone et al., 2009). Among these, visual dysfunction—particularly dry eye disease (DED)—constitutes a frequent yet underdiagnosed complaint (Biousse et al., 2004; Nieto-Escamez et al., 2023). Our findings reaffirm that DED is not merely a comorbid condition but an integral non-motor manifestation of PD, with a prevalence reaching 70%, consistent with recent meta-analyses (Nagino et al., 2022). This high prevalence underscores the necessity of integrating ocular surface evaluation into the routine assessment of PD patients.

### 4.2 Dry eye in PD: current findings and proposed mechanisms

We observed significantly worse DED-related parameters in PD patients, including elevated OSDI scores, reduced Schirmer I test values, shortened BUT, and higher corneal fluorescein staining relative to controls. These results align with prior clinical studies (Biousse et al., 2004; Nagino et al., 2022) and extend the work of Marino et al. (2020) and Nagino et al. (2022), who reported dysfunctional blink mechanics and autonomic impairment contributing to tear film instability.

Notably, the strong positive correlations between DED severity and both disease duration and LEDD suggest that dry eye may progress in parallel with dopaminergic degeneration. The association between PD progression and DED may be mediated by several pathophysiological mechanisms. These include degeneration of brainstem nuclei, such as the locus coeruleus and dorsal vagal nucleus, which disrupts autonomic control of lacrimal secretion; reduced corneal sensory innervation due to trigeminal neuropathy, which impairs afferent signaling within the lacrimal functional unit (Jankovic, 2008); and potential systemic neuroinflammatory processes that adversely affect ocular surface tissues. Collectively, our data support the concept that PD-related DED is not solely a superficial ailment but a neurologic disorder manifesting at the ocular surface.

### 4.3 Pathological mechanisms of DED and CET characteristics in PD

While the neurodegenerative process of Parkinson’s disease (PD) has been extensively studied in the central nervous system, its manifestations within the ocular system provide a unique window into disease progression. Notably, involvement of the posterior segment is well-documented. Recent research using optical coherence tomography (OCT) has demonstrated that patients with PD exhibit specific thinning of the retinal nerve fiber layer (RNFL) and ganglion cell complex (GCC), which correlates with disease duration and severity (Kakimoto et al., 2025). This finding offers an intriguing parallel to our observation of progressive corneal epithelial thinning in the peripheral zones, suggesting a coordinated yet topographically distinct impact of PD neurodegeneration on ocular tissues. To investigate this anterior segment manifestation further, we examined the specific interplay between dry eye disease (DED) and corneal epithelial thickness (CET) in PD.

Evidence from various disease models indicates that alterations in corneal epithelial thickness (CET) are closely associated with ocular surface health across different dry eye disease (DED) subtypes. In aqueous-deficient DED, such as in Sjögren’s syndrome, widespread epithelial thinning has been correlated with reduced tear secretion and inflammatory mediators (Gupta et al., 2022). Similarly, in evaporative DED associated with meibomian gland dysfunction, irregular epithelial thickness distribution reflects chronic epithelial stress and altered differentiation (Abou Shousha et al., 2020). These observations highlight CET as a sensitive structural biomarker that often correlates with functional deficit. Thus, CET mapping offers insights into ocular surface health that extend beyond conventional tear metrics.

Building upon this foundation, emerging evidence suggests that neurodegenerative conditions, particularly Parkinson’s disease (PD), may present distinct CET alteration patterns attributable to their unique pathophysiology. While the previously mentioned DED subtypes primarily involve inflammatory or glandular mechanisms, PD-related DED may stem from central neurodegenerative processes affecting peripheral neural pathways (Armstrong, 2015). This distinct neuropathic component differentiates PD-related CET changes from those observed in conventional DED, as evidenced by the topographic thinning pattern identified in our study.

Our findings provide substantive evidence for PD-specific CET alterations, demonstrating significant thinning in the temporal and nasal corneal quadrants in PD patients compared to controls. This topographic pattern—predominantly affecting the peripheral cornea—aligns with the “length-dependent degeneration” characteristic of PD neuropathy (Marogianni et al., 2020), as longer trigeminal nerve fibers innervating the corneal periphery are more vulnerable to axonal damage (Jankovic, 2008).

The correlation between temporal CET and BUT (*r* = 0.164, *p* = 0.024) further supports the functional significance of these structural changes. This relationship suggests that neuropathic epithelial thinning may exacerbate tear film instability through altered surface regularity and compromised epithelial barrier function. The integrity of the corneal epithelium is essential for maintaining ocular surface homeostasis and tear film stability (De Silva et al., 2019). Notably, the concentric pattern of CET thinning in PD differs from the more diffuse thinning observed in conventional aqueous-deficient DED, potentially reflecting its neuropathic rather than inflammatory origin.

These findings position CET mapping as a promising tool for identifying PD-related ocular surface pathology. The distinct topographic pattern of epithelial thinning may serve as a morphological signature that differentiates PD-related DED from other subtypes, while its correlation with disease parameters suggests potential utility in monitoring progression. Future studies comparing CET patterns across different DED etiologies could further elucidate the specificity of these findings for neurodegenerative conditions.

### 4.4 Dynamic association between CET and DED parameters

Building upon the characterization of PD-specific CET alterations, this study utilized AS-OCT to systematically evaluate CET distribution patterns and their correlation with DED parameters in Parkinson’s disease. To our knowledge, this is the first report to identify a distinct topographic pattern of epithelial thinning—predominantly in the temporal, nasal, and superior 6-mm corneal quadrants—and to establish a significant positive correlation between temporal CET and BUT (ρ = 0.235, *p* = 0.027).

Notably, although no direct association was observed between zonal CET values and OSDI scores (*p* > 0.05), the identified correlation with BUT suggests that structural epithelial changes are more closely linked to objective functional impairments than to subjective symptoms in PD. This finding implies that reduced tear film stability may drive peripheral corneal epithelial remodeling through mechanical stress and oxidative damage (Gupta et al., 2022).

Furthermore, degeneration of the temporal corneal nerve plexus—which serves as the afferent limb of the lacrimal secretory reflex—may disrupt neurogenic stimulation of tear secretion, providing a plausible mechanism for the concomitant BUT reduction and temporal CET thinning (Misra et al., 2017). This neurotrophic failure likely contributes to a vicious cycle comprising epithelial damage, tear film instability, and further neural impairment.

The concentric 6-mm CET mapping may thus serve as a novel non-invasive biomarker for evaluating tear film function and neurodegeneration severity in PD. However, future studies with larger cohorts are warranted to establish diagnostic thresholds and validate its clinical utility.

### 4.5 Disease progression (H-Y Stage) and dynamic corneal structural changes

The H-Y staging system serves as a key clinical framework for classifying motor symptom severity and functional impairment in Parkinson’s disease (PD), with higher stages reflecting more extensive neurodegeneration and disability (Hoehn and Yahr, 1967). In the present study, although corneal epithelial thickness (CET) differences across H-Y stages did not consistently reach statistical significance—likely due to sample size constraints—a clear trend of progressive epithelial thinning was observed from stage 1 to stage 3 across multiple corneal zones. For instance, all quadrants within the concentric 5- and 6-mm peripheral zones exhibited a trend of progressive corneal epithelial thinning with advancing H-Y stage. Conversely, central corneal thickness demonstrated a slight but significant increase in stage 3, suggesting a potential compensatory mechanism.

This spatial discrepancy—progressive peripheral thinning accompanied by central thickening—may mirror distinct neurodegenerative and adaptive processes across PD stages. In early disease, peripheral corneal thinning may arise from length-dependent degeneration of trigeminal sensory nerve fibers, which traverse longer distances and are particularly vulnerable to axonal damage, leading to impaired neurotrophic support and epithelial atrophy (Jankovic, 2008; Marogianni et al., 2020). In advanced stages, central thickening could be driven by chronic inflammatory processes (e.g., elevated IL-6 and TNF-α) or reactive epithelial remodeling mechanisms attempting to restore barrier integrity (Marogianni et al., 2020). Chronic inflammation has been demonstrated in both the central nervous system and peripheral tissues in Parkinson’s disease, potentially contributing to tissue remodeling (Hirsch and Hunot, 2009).

Consistent with the deterioration in corneal structure, dry eye parameters also displayed progressive worsening with higher H-Y stages: OSDI scores increased (46.59–63.63, *p* = 0.057), while BUT declined (5.4 s–4.79 s, *p* = 0.419). These findings support the clinical trajectory wherein motor dysfunction—including reduced blink rate and completeness—aggravates ocular surface exposure, tear film instability, and epithelial damage. The correlation between corneal sensitivity, blink rate, and tear metrics reported by Demirci et al. further reinforces the integral role of corneal innervation in regulating tear film homeostasis (Misra et al., 2017).

Although the current cross-sectional analysis did not detect statistically significant CET differences across all H-Y subgroups, the identified trends align with established models of PD-related neuropathy and disease progression. Specifically, the pattern of zonal epithelial thinning may provide a quantifiable reflection of progressive neurodegeneration, offering a potential objective correlate to clinical staging.

In conclusion, corneal epithelial thickness mapping—particularly within the peripheral zones—may emerge as a complementary tool for monitoring PD progression and severity. Future longitudinal studies with larger cohorts are warranted to validate CET as an auxiliary biomarker for H-Y staging and to clarify the molecular mechanisms underlying corneal structural changes in PD.

### 4.6 Clinical implications and study limitations

AS-OCT offers a novel approach for early monitoring of PD-related ocular surface disorders. Corneal epithelial thickness mapping can potentially quantify disease progression (e.g., dynamic changes with H-Y stage) and assess dopaminergic treatment efficacy (Gupta et al., 2022). The search for reliable, non-invasive biomarkers for Parkinson’s disease progression remains an important research priority (Mollenhauer et al., 2014).However, this study has several limitations that should be acknowledged.

The cross-sectional design limits causal inference between PD progression and corneal changes, though the associations observed are biologically plausible and align with established neuropathic models (Jankovic, 2008; Armstrong, 2015). A longitudinal study is planned to better assess temporal dynamics. The moderate sample size (*n* = 120 eyes) may have reduced power to detect weak effects; however, *post hoc* power analysis confirmed sufficient power (> 80%) for primary outcomes, and effect estimates are provided to facilitate future meta-analysis. Multicenter expansion of the cohort is ongoing. Finally, the absence of direct measures of corneal nerve density or inflammatory cytokines limited mechanistic insight. Nonetheless, clinical correlations with H-Y stage and tear parameters indirectly support roles of neurodegeneration and inflammation (Marogianni et al., 2020; Rahman et al., 2021). Future work will incorporate corneal confocal microscopy and tear cytokine profiling to clarify molecular pathways

## 5 Conclusion

This study utilized AS-OCT to measure CET in various corneal zones of PD patients. The observed changes in corneal epithelial thickness were correlated with patients’ H-Y stage, suggesting its potential utility in disease staging. Furthermore, significant thinning was identified specifically in the concentric 6-mm zone, which exhibited a positive correlation with BUT. These findings indicate that CET within the concentric 6-mm zone may serve as a novel biomarker for assessing tear film function in PD, although its diagnostic thresholds and clinical significance require validation in larger cohorts. We anticipate that these findings will be supported by future prospective studies with larger participant numbers.

## Data Availability

The raw data supporting the conclusions of this article will be made available by the authors, without undue reservation.
